# Three-dimensional visualization of lithium metal anode via low-dose cryogenic electron microscopy tomography

**DOI:** 10.1016/j.isci.2021.103418

**Published:** 2021-11-09

**Authors:** Xiangyan Li, Bing Han, Xuming Yang, Zhipeng Deng, Yucheng Zou, Xiaobo Shi, Liping Wang, Yusheng Zhao, Sudong Wu, Meng Gu

**Affiliations:** 1 Academy for Advanced Interdisciplinary Studies, Southern University of Science and Technology, Shenzhen 518055, China; 2 Department of Materials Science and Engineering, Southern University of Science and Technology, Shenzhen 518055, China; 3 Department of Materials, Imperial College London, London SW7 2AZ, UK; 4 Guangdong Provincial Key Laboratory of Energy Materials for Electric Power, Southern University of Science and Technology, Shenzhen 518055, China; 5 Guangdong-Hong Kong-Macao Joint Laboratory for Photonic-Thermal-Electrical Energy Materials and Devices, Southern University of Science and Technology, Shenzhen 518055, China; 6 Key Laboratory of Energy Conversion and Storage Technologies, Southern University of Science and Technology, Ministry of Education, Shenzhen 518055, China

**Keywords:** Energy materials, Materials characterization, Materials characterization techniques, Materials chemistry, Materials science

## Abstract

The structure of lithium (Li) metal anode, including the Li metal and the solid electrolyte interphase (SEI), is critical to the investigation of cycle stability or decay mechanisms. The three-dimensional (3D) visualization of Li metal and SEI, however, has not been demonstrated yet, owing to the lack of 3D characterization techniques and the susceptibility of Li metal anode toward oxygen, moisture, as well as electron beam. Herein, we introduce a successful 3D presentation of deposited Li metal and SEI established via low-dose cryogenic electron microscopy tomography. The Li metal anode is imaged in low-dose mode at different tilt angles and then aligned and reconstructed into a 3D image through an expectation-maximization algorithm. The spherical Li deposits and SEI are confirmed in the 3D tomography of Li metal anode. It is also discovered that the Li metal corrodes and SEI turns concave owing to possible self-discharge after long-time rest.

## Highlights

• We achieved 3D characterization of Li metal and SEI using cryo-STEM tomography
• The uniformity and local thickness of the SEI can be accurately measured in 3D
• The Li metal corrodes and turns concave due to self-discharge after deposition

## Graphical abstract

**Figure:**
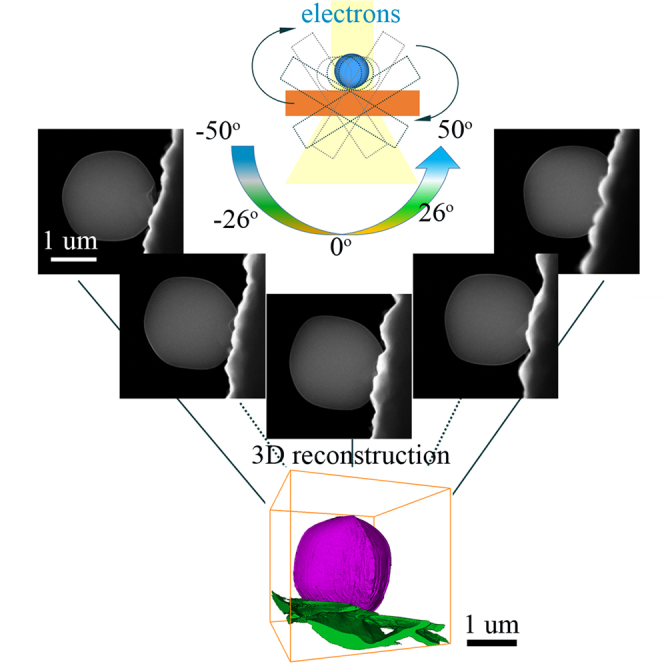

## Introduction

The rapid growth of electric vehicle markets and smart grid implementation creates an urgent need for developing batteries with high energy density and long calendar life (Alper, 2002; Dunn et al., 2011). The energy density of commercial lithium (Li)-ion batteries has been significantly improved in the past decades, and it is already very close to their ceiling values (Yang et al., 2020). Further substantial increase in energy density will necessarily depend on innovation on new electrode materials, and one typical case is Li metal anode (Shi et al., 2018; Zhang et al., 2018). The replacement of graphite anode with Li metal will undoubtedly help to achieve higher energy density, and it will also allow the use of Li-free cathode materials to make batteries with much higher capacity, such as lithium-sulfur batteries (Jiang et al., 2021; Lin et al., 2021; Manthiram et al., 2014). Dendrite formation is the major drawback of Li metal anode, which could penetrate separator and cause internal short circuit (Liu et al., 2017; Yang et al., 2019). The high reactivity of Li is also a very disturbing feature, which could cause side reactions and lead to loss of Li during operation (Lin et al., 2019). Solid electrolyte interphase (SEI), which is usually formed during initial cycles, can passivate the surface of Li metal but allow the passage of Li ions (Goodenough and Kim, 2009; Peled et al., 1995).

Electron microscopy (EM), including transmission electron microscopy (TEM) and scanning transmission electron microscopy (STEM), are generally used to directly observe the morphology of Li deposits and SEI (Cheng et al., 2020; Han et al., 2021; Li et al., 2017, 2018; Zachman et al., 2018). During EM characterizations, beam damage occurs. Electrons with high energy knock on samples, leading to the displacement of atoms and further creating point defects in a crystal. Besides, the inelastic scattering of electrons transfers heat and increases phonons, leading to the increase of the temperature of a sample (Egerton et al., 2004). The Li deposits and SEI are very sensitive to electrons and could be easily damaged by the electron beam (Li et al., 2017, 2018). Thus, the conventional EM is quite limited in imaging the structure of such materials. The low-dose cryogenic electron microscope (cryo-EM) is the best choice for characterizing high-electron-sensitive materials (Wang et al., 2017, 2018). On the one hand, it can sustain the native state of the sample by cooling down the sample and maintaining the temperature at 80 K. On the other hand, a low-dose technique can significantly decrease the electron dosage because it captures a STEM image with several hundred rather than tens of thousands of electrons per square angstrom (the general electron dosage in conventional STEM) (Li et al., 2017). Using low-dose cryo-EM, the influence of the type of electrolytes on the growth of Li dendrites, the nanostructure of SEI, and the stable nature of the SEI were studied (Cheng et al., 2020; Han et al., 2021; Li et al., 2017, 2018; Zachman et al., 2018). However, in most reported papers, studies were focused on two-dimensional (2D) characterizations. It should be noticed that the TEM and STEM images are 2D projections of a three-dimensional (3D) structure, and the information in the Z direction is accumulated (Li et al., 2020). Thus, it is hard to know the complex 3D intrinsic structures from 2D images.

In this study, a method for characterizing the 3D intrinsic structure of high-beam-sensitive samples was established based on low-dose cryo-EM tomography. To be specific, by optimizing the sample preparation process, the structure of Li deposits and the SEI layer were well kept. By using cryo-EM, projections of Li deposits and SEI viewed from −50° to 50° were acquired without obvious electron damage. Through an expectation-maximization algorithm, the 3D structure was reconstructed. Using this method, 3D morphologies and the morphological evolution of Li deposits and SEI layer that formed in Li||Cu coin cells were characterized.

## Results

### 3D characterization method of Li deposits and SEI

**Figure 1:**
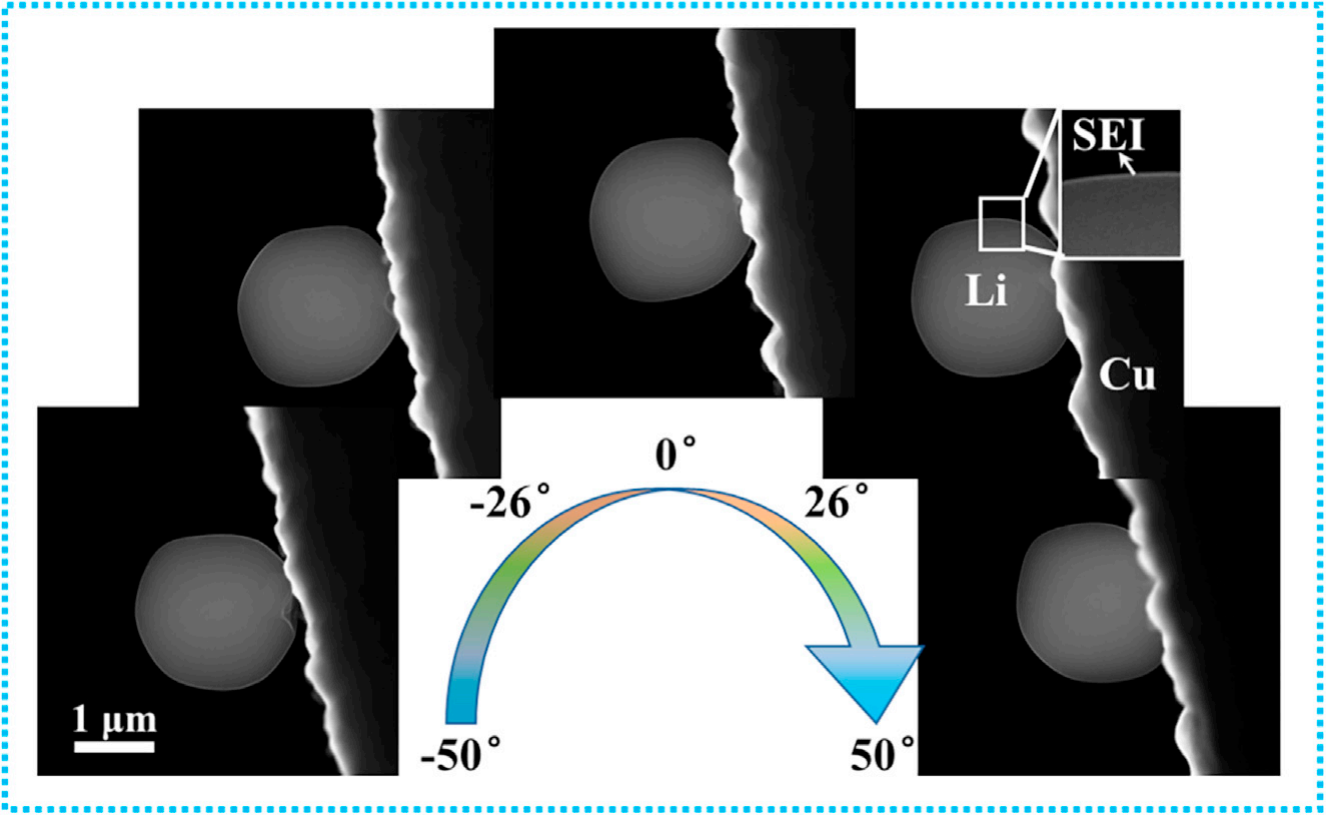
Representative STEM projections of the Li metal anode with tilt angle of −50°, −26°, 0°, 26°, and 50° (See also Video S1 to view the Li metal anode at different angles).

Figure 1 and Video S1 show the STEM projections of the Li metal anode, including the deposited Li and the SEI layer, captured at different tilt angles. Since Li deposits and SEI are very vulnerable to oxygen, moisture, and electron beam, specimens were carefully prepared in an Ar-filled glove box and transferred under cryogenic condition to minimize air exposure time as much as possible. During the EM tomography characterization, a low-dose imaging technique was applied to reduce beam damage. A high-angle annular dark field (HAADF) detector was used to detect electrons. The specimen was cooled by liquid nitrogen during the whole imaging process, which could offset the heat generated by inelastic scattering of electrons under the beam radiation. It should be emphasized that the total electron exposure time for EM tomography characterization is much longer than conventional 2D imaging, because the region of interest should be imaged for dozens of times to acquire projections at tilt-series angles. At the same time, the tilt range should be wide enough to acquire information for 3D reconstruction and the tilt step should be small, which would add the total exposure time. The parameters, such as tilt range and step, exposure time, and resolution, should be carefully checked, to ensure that no noticeable damage occurs. In this work, the structure of Li deposits stays unchanged under beam radiation during the whole imaging process (Figure 1), that is, the total electron-induced damage is not enough to cause obvious deformation of Li deposits and SEI.

Video S1. STEM projections of the Li metal anode acquired at different tilt angles (the tilt angle increases from −50° to +50° with an increasement of 2°), related to Figure 1

**Figure 2:**
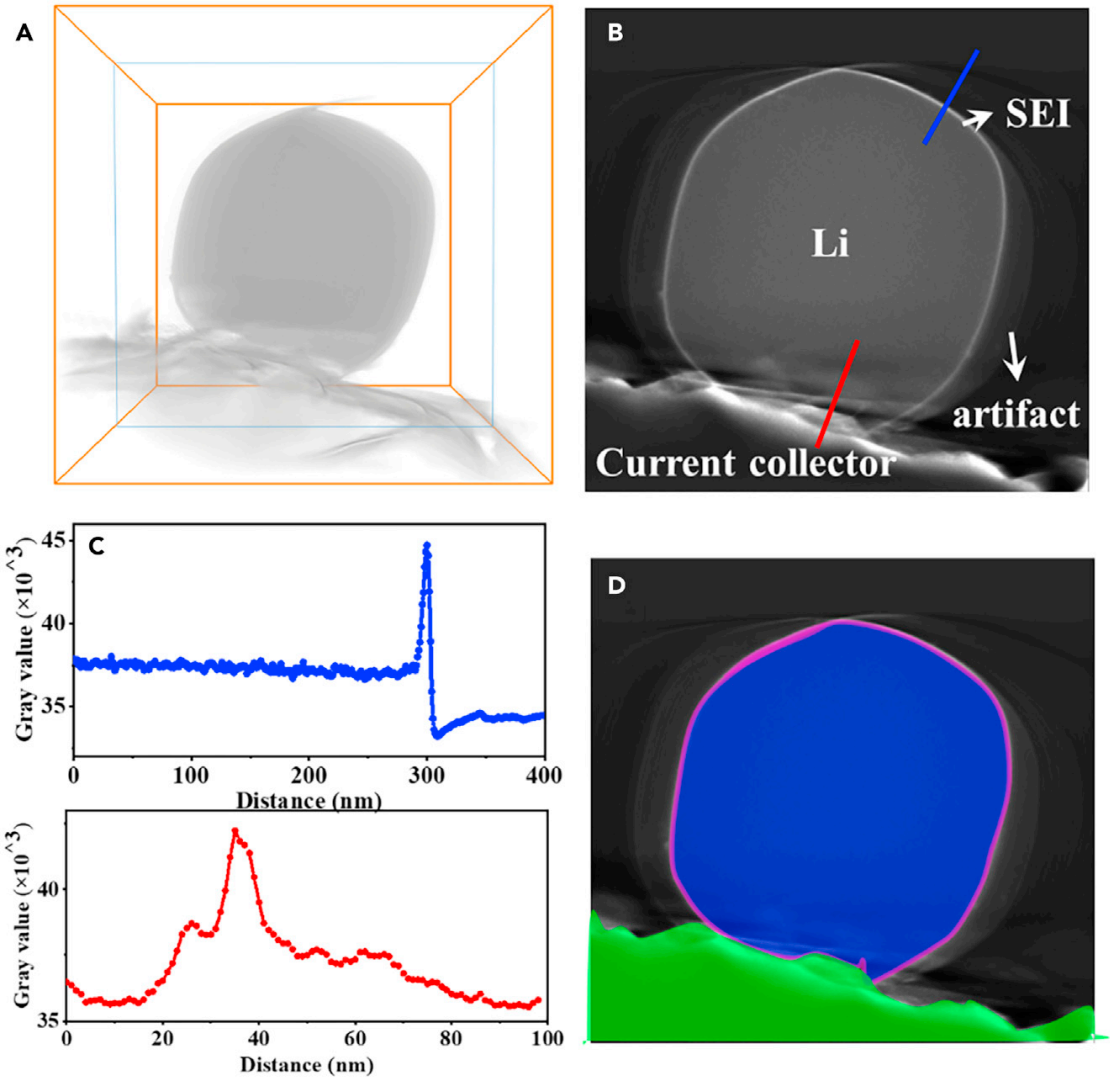
The method introduction for identifying Li metal, SEI layer, and Cu current collector (A) 3D reconstruction of Li metal anode (please see Video S2 to view the volume render process). (B) An orthogonal slice of the 3D image. (C) The corresponding gray value-distance curve from Li deposition to vacuum across SEI (shown by the blue line in B) and gray value-distance curve from Li deposition to the current collector (shown by the red line in B). (D) The orthogonal slice that is colored by blue (Li deposition), purple (SEI layer), and green (Cu current collector), according to gray values of Li metal, SEI, and current collector.

Figure 2A and Video S2 show the 3D reconstruction of the Li metal anode, which is made up of 512 × 512 × 512 voxels (the voxel size is 3.9 nm) with different brightness. The brightness of voxels is defined by giving a gray value to each voxel, ranging from 1 to 2^16^. The ortho slice of the 3D reconstruction of Li metal anode is shown in Figures 2B and 2D, and the corresponding gray value-distance curve from Li deposition to vacuum across SEI (shown by the blue line in Figure 2B) and gray value-distance curve from Li deposition to the current collector (shown by the red line in Figure 2B) are shown in Figure 2C. The gray value of the SEI (38,000–44,000) is higher than that of the Li metal (37,000–38,000), because the SEI contains elements heavier than Li, such as O and F, which cause more elastic scattered electrons to be detected by the detector (see Figure S1). The area with much lower gray value (33,000–34,500) represents vacuum. By hiding the voxels derived from vacuum area, namely, the volume render process, the morphology of the deposited Li, SEI, and current collector (with voxel intensity of 35,500–38,500) can be visualized as shown in Video S2. Based on the positions, gray value, and shape of components in the 3D image, Li metal, SEI, and current collector are distinguished and semi-automatically colored to be blue, purple, and green, respectively (Figure 2D and Video S3). It should be pointed out that there exist some rings with low gray value in the outer ring of the SEI in Figure 2A, but actually, these structures do not exist (see Figure 1). These rings are called artifacts in the 3D image, as the specimen was not tilted in a full range during the acquisition of STEM projections, and information from −90° to −50° and 50°–90° was lacking. Nevertheless, the contrast between vacuum and Li metal or SEI is sharp, and labeling of the various components in 3D images is reliable. By presenting Li metal, SEI, and current collector in different color, the 3D image of the Li metal anode could be more comprehensible.

Video S2. 3D reconstruction of Li-metal anode, related to Figure 2

**Figure 3:**
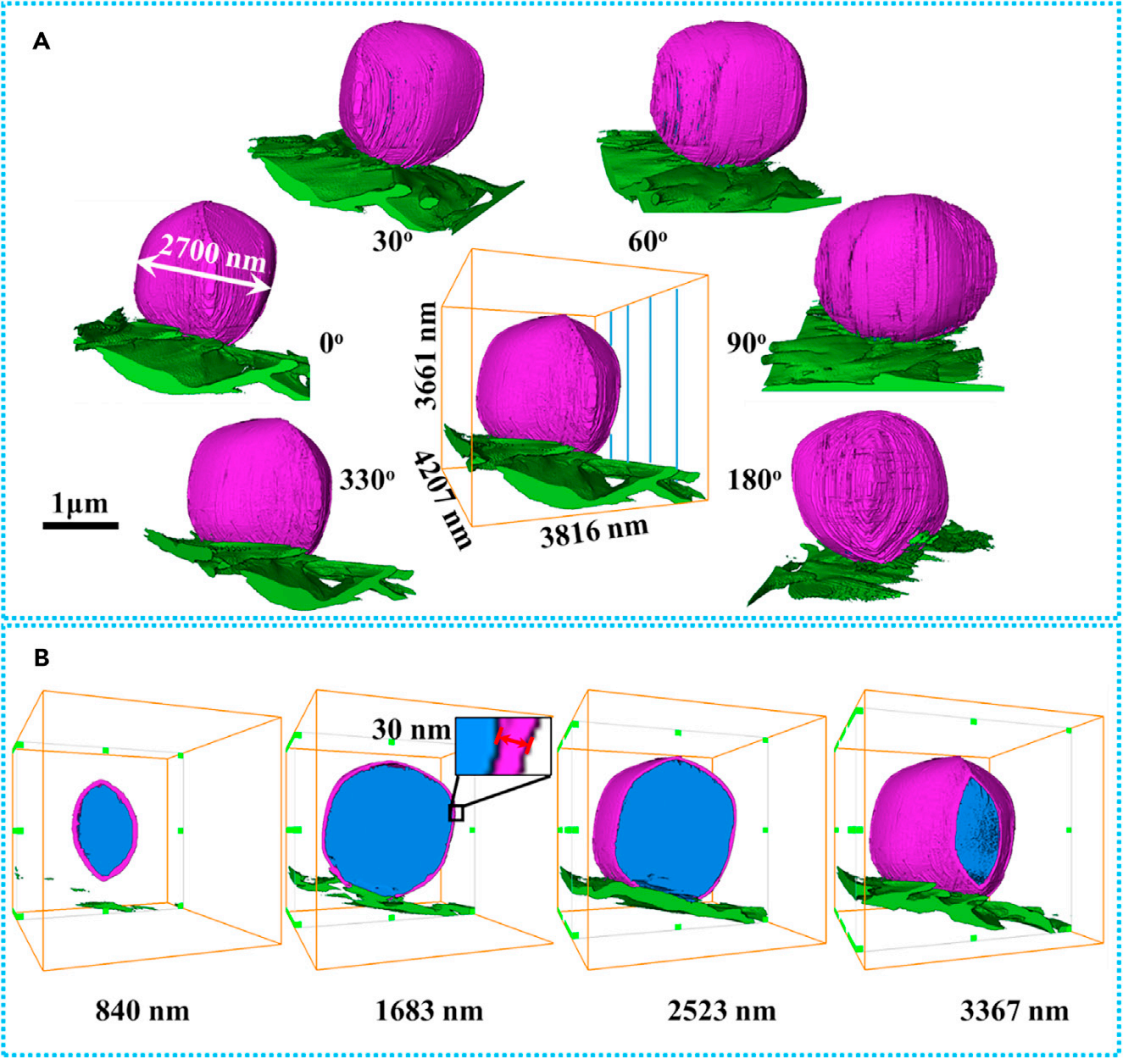
Colored 3D reconstruction of Li-metal anode: Li deposition (blue), SEI (purple), and current collector (green) (A) Images of Li metal anode viewed from different angles. (B) Cross-sectional views at different depths (840, 1 683, 2,523, and 3,367 nm). 3D visualization is available in Videos S3 and S4. Panel B share the same scale bar with Panel A.

Video S3. Colored 3D reconstruction of Li-metal anode viewed from different angles, related to Figure 3

### The 3D intrinsic structure of Li deposits and SEI

Figure 3A shows the colored 3D image of Li metal anode acquired at various selected tilt angles, and the animation showing the image in continuous angles is provided as Video S3 (the voxel size is 5.5 nm). Figure 3B shows the cross sections at selected depth (the value in z axis is 840, 1,683, 2,523, and 3,367 nm), and the cross sections at continuous depth are shown in Video S4. The deposited Li metal is roughly spherical with a diameter about 2.7 μm, and it is conformably coated by a thin SEI layer with a thickness about 30 nm.

Video S4. Cross sections of colored 3D reconstruction of Li-metal anode viewed from different depths, related to Figure 3

**Figure 4:**
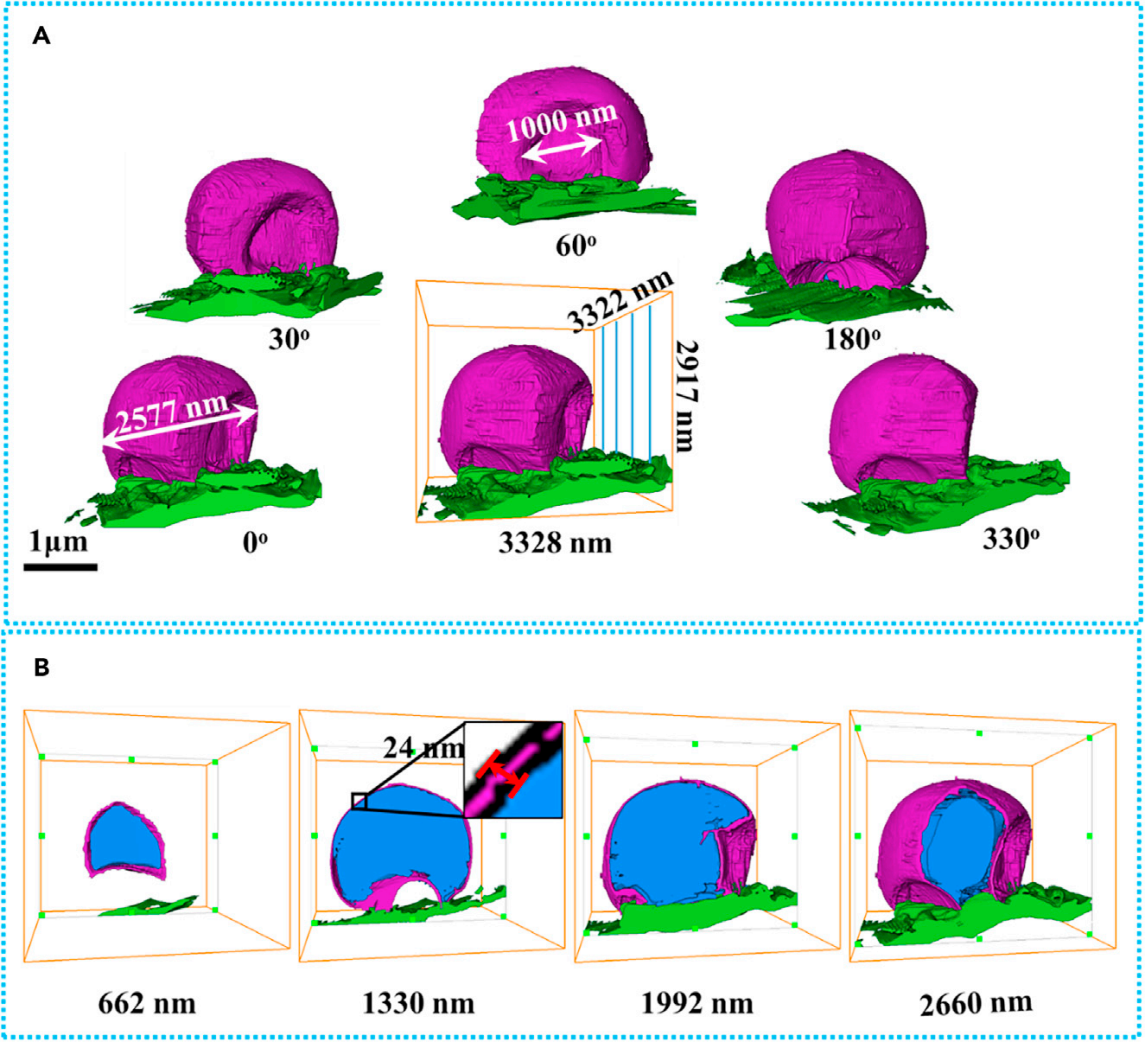
Colored 3D reconstruction of Li-metal anode after placing the Cu-TEM-grid in the coin cells for 10 h: Li deposition (blue), SEI layer (purple), and Cu current collector (green) (A) 0°–330° view showing the Li-metal anode viewed from different angles. (B) Cross-sectional views at different positions of z axis (662, 1,330, 1,992, and 2,660 nm). 3D visualization is available in Videos S6 and S7. Panel B share the same scale bar with Panel A.

To characterize the morphologic change of Li metal during the rest time, the TEM grid deposited with Li is taken out for cryo-EM imaging after the cell is aged for 10 h. Video S5 shows the STEM projections of the Li metal anode captured at different tilt angles. Figure 4A and Video S6 show the colored image of Li metal anode at different angles. The particles with high contrast around the Li metal anode in Video S5 are ice particles. They used to float above the liquid nitrogen and then absorbed onto samples when the TEM grid is immersed in liquid nitrogen in the STEM sample preparation. These particles are not the structure that we focused on. As a result, they were not colored. The striking difference is the concave of the Li metal anode after aging, which is probably due to the corrosion of the Li metal inside. The cross sections at different depths are shown in Figure 4B, and it is clearly discerned that there are internal voids inside the surface SEI layer (see also Video S7). This phenomenon indicates that Li metal is not untouched but corroded during the rest time, and measures should be taken in building practical Li metal batteries with little excess lithium.

Video S5. STEM projections of the Li metal anode after placing the Cu-TEM-grid in the coin cells for 10 h, the tilt angle increases from −50° to −50° with an increasement of 2°, related to Figure 4

Video S6. Colored 3D reconstruction of Li-metal anode after placing the Cu-TEM-grid in the coin cells for 10 h, related to Figure 4

Video S7. Cross-sectional views of colored 3D reconstruction of Li-metal anode viewed from different depths after placing the Cu-TEM-grid in the coin cells for 10 h, related to Figure 4

## Discussion

**Figure 5:**
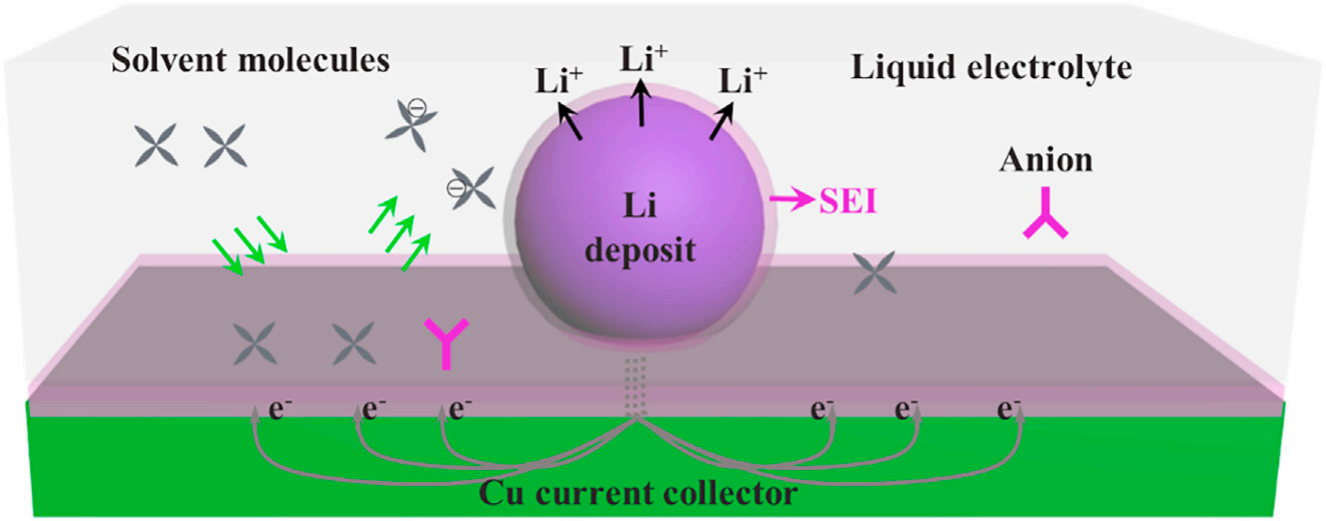
Schematic of the proposed Li corrosion process

It has been previously reported by Lin et al. that Li could corrode through a galvanic process between Li and current collector (Lin et al., 2019). We proposed a similar corrosion mechanism based on possible self-discharge, as illustrated in Figure 5. The Li metal and the electrolyte composed a micro battery. The overall process is the side reaction between Li metal and the electrolyte. But Li metal is oxidized into Li^+^ on SEI and released into the electrolyte, and electrolyte is reduced on the surface of the Cu current collector. The electron conduction pathway is possibly from Li metal to the Cu current collector.

In this study, 3D structures of Li metal anode, which is vulnerable to air exposure and beam sensitive, is imaged and visualized with low-dose cryo-EM tomography. Through the images viewed from different angles or cross sections, the 3D distribution details of Li metal, SEI, and current collector are provided. Spherical Li deposits and SEI are observed in fresh Li metal anode, but for the one rested for 10 h, internal voids emerge and SEI turns concave. The spatial characteristics of active electrode materials and electrode-electrolyte interphases could be critical for researchers to understand the entire electrode process, find underlying failure mechanisms, and ultimately, give strategic solution to prolong calendar life of batteries. We hope this work could draw peer researchers' interest to the practicality of 3D visualization of electrodes, and more fruitful outcome can be expected from this technique.

### Limitations of the study

In this work, only the overall 3D structure of the Li deposition and the SEI layer before and after the cell being aged for 10 h was characterized. However, the effect of aging on the crystallography of the Li formation as well as the composition of SEI was not studied in more depth.

## STAR★Methods

### Key resources table

**Table:** 

REAGENT or RESOURCE	SOURCE	IDENTIFIER
**Chemicals, peptides, and recombinant proteins**		

Li metal	DoDo Chem	http://dodochem.net/
Electrolyte	DoDo Chem	http://dodochem.net/
Cu foil	canrd	http://www.canrd.com/shop/product/list?productCategoryId=d368533d5fbd4b8db6c673069fbdaab8
DOL	DoDo Chem	http://dodochem.net/
copper grid	TED PELLA	https://www.tedpella.com/grids_html/gilder.htm#anchor1540234

**Software and algorithms**		

DigitalMicrograph	Gatan Inc	https://www.gatan.com/
Inspect 3D		https://www.thermofisher.com/order/catalog/product/INSPECT3D?SID=srch-srp-INSPECT3D
Avizo	Thermo fisher scientific	www.thermofisher.com/amira-avizo
Expectation-maximization algorithm		https://people.duke.edu/∼ccc14/sta-663/EMAlgorithm.html

**Other**		

Cryo TEM	Thermofisher Inc	https://www.thermofisher.cn/cn/zh/home.html

### Resource availability

#### Lead contact

Further information and requests for resources and reagents should be directed to and will be fulfilled by the lead contact, Meng Gu (gum@sustech.edu.cn)

#### Materials availability

This study did not generate new unique reagents

### Method details

#### Sample preparation

The TEM specimen of Li metal anode is prepared by directly depositing Li on a naked TEM grid in a coin-type Li||Cu cell. The electrolyte is 1.0 M lithium bis(trifluoromethane)sulfonamide (LiTFSI) in 1,3-dioxolane/1,2-dimethoxyethane (DOL:DME = 1:1 vol/vol) with 1% LiNO_3_ (purchased from DoDo Chem., China). The TEM grid is placed on the top of copper current collector. The Li deposition is realized by applying a constant current of 0.5 mA cm^−2^ to the Li||Cu cell for 1 h. The TEM grid is taken out immediately from the cell in Ar-filled glovebox when the Li deposition is completed and washed by being immersed in DOL for a few seconds to remove adsorbed salts. The TEM grid is dried by a heating stage at 343 K for 10 min. To minimize air exposure during the specimen transfer from glovebox to TEM column, the TEM grid is sealed in an airtight container and immersed in liquid nitrogen, the TEM grid transfer from the container to a cryo-holder (Fischione) is also under liquid nitrogen, and a built-in shutter enables the cryo-holder insertion without air exposure of the TEM grid. For the sample aimed at the investigation of Li metal corrosion, the cell is aged for 10 h before the TEM grid is taken out.

#### Electron tomography characterization

The image of deposited Li and SEI is acquired by a Cryo-TEM (Titan Krios G3i D3845, FEI, America) at 300 kV. The holder is tilted from −50° to +50° with a step of 2°. At each tilting angle, an STEM projection is captured. The dwell time is 2 μs, and the magnification is 14,000. To minimize the beam damage, the electron dosage is controlled at ∼200 e/nm^2^ and the temperature is maintained at 80 K. A HAADF detector is used to record electron signals using a convergence semi-angle of 25 mrad. The resolution of acquired images is 2048 × 2048, and the logical depth is 2^16^. The sequential tilt-series images are aligned using Inspect 3D software (Ver. 4, FEI, America), and the 3D structure of the Li deposits is reconstructed through an expectation maximization (EM) algorithm with the iteration time of 25 (Noumeir et al., 1995). The EM algorithm is an iterative optimization method to find maximum likelihood estimates of parameters in statistical models. The gray values of voxels in 3D-STEM images are estimated according to the gray values and angles of tilt-series images. Then, the missing information are estimated according to the current information. Repeat this process until the final convergence and the iteration is over. The 3D reconstruction is then post-processed, colored, and volume rendered with Avizo software (Ver. 9.0.1, FEI, America).

## Data Availability

• Private datasets utilized in this study are available from the lead contact upon reasonable cooperation request. • The paper does not report original code.
