# Supplementary material for: Three-dimensional visualization of lithium metal anode via low-dose cryogenic electron microscopy tomography
Source: iScience. 2021 Nov 9;24(12):103418. doi: 10.1016/j.isci.2021.103418 (PMC8633965; doi:10.1016/j.isci.2021.103418)
Supplement: Document S1. Figure S1 [file mmc1.pdf]

iScience, Volume *24*

Supplemental information

Three-dimensional visualization of lithium metal anode via low-dose cryogenic electron microscopy tomography

Xiangyan Li, Bing Han, Xuming Yang, Zhipeng Deng, Yucheng Zou, Xiaobo Shi, Liping Wang, Yusheng Zhao, Sudong Wu, and Meng Gu

Supplemental Information

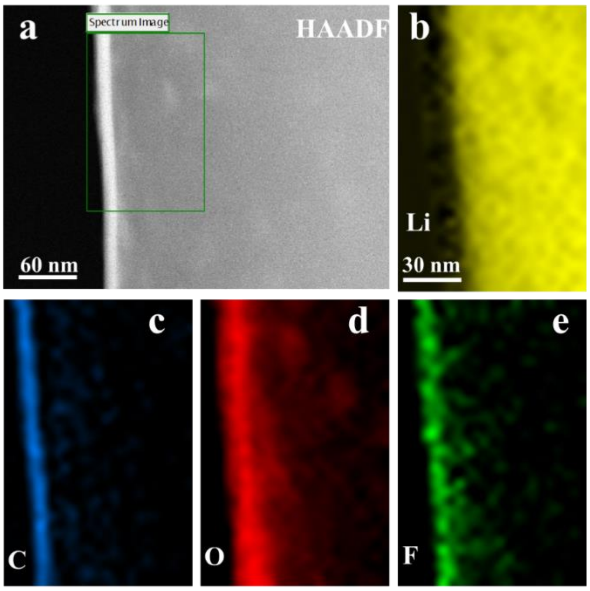

Figure S1. Elemental analysis of Li deposit and SEI layer. (a) HAADF image of Li deposit and SEI layer; cryo-electron energy loss spectroscopy (EELS) mapping of (b) Li, (c) C, (d) O, and (e) F (Figure c-e shares the same scale bar with Figure b). **Related to Figure 2.**
